# Quality of Life (QoL) among medical students in Saudi Arabia: a study using the WHOQOL-BREF instrument

**DOI:** 10.1186/s12909-019-1775-8

**Published:** 2019-09-09

**Authors:** Husam Malibary, Mohammad M. Zagzoog, Maysaa A. Banjari, Ryan O. Bamashmous, Anoud R. Omer

**Affiliations:** 1 Department of Internal Medicine, Faculty of Medicine, King Abdulaziz University, Jeddah, Saudi Arabia; 2 Department of Surgery, King Abdulaziz Medical City, Jeddah, Saudi Arabia; 3 Department of Pediatrics, Faculty of Medicine, King Abdulaziz University, Jeddah, Saudi Arabia; 4 Clinical Research Unit, King Abdulaziz University Hospital, Jeddah, Saudi Arabia; 5 Medical Education Department, Faculty of Medicine, King Abdulaziz University, Jeddah, Kingdom of Saudi Arabia

**Keywords:** Medical students, Medical education, Quality of life (QoL), Grade point average (GPA)

## Abstract

**Background:**

Poor Quality of Life (QoL) among medical students is associated with an unhealthy lifestyle, psychological distress, and academic failure, which could affect their care for patients in the future. This study aimed to evaluate the reliability and validity of the Arabic WHOQOL-BREF tool among Saudi medical students and to assess the effect of gender, educational level, and academic performance on their QoL.

**Methods:**

This was a cross-sectional study among medical students of King Abdulaziz University in February 2016, using the Arabic version of the WHOQOL-BREF instrument.

**Results:**

Six-hundred-thirty medical students were included, where females constituted (51.1%). Cronbach’s α coefficient for the overall domains of WHOQOL-BREF was 0.86. Students’ self-reported QoL mean score was 3.99 ± 0.95, and their mean score for the overall satisfaction with health was 3.66 ± 1.06. The environmental domain had the highest mean score (67.81 ± 17.39). High achievers showed lower psychological health, while poor academic performance was associated with better psychological health and social relationship QoL scores (*P* < 0.013 and *P* < 0.014, respectively).

**Conclusions:**

The WHOQOL-BREF is valid and reliable for assessing QoL among Saudi medical students. Although gender and academic year had no impact on the students’ QoL, better-performing students reported lower psychological health and social relationships scores.

## Background

The World Health Organization (WHO) defined Quality of Life (QoL) as “an individual’s perception of their position in life, in the context of the culture and value systems in which they live, and in relation to their goals, expectations, standards and concerns” [1]. QoL is comprised of multiple aspects, including psychological health, physical well-being, social relationships, and environmental conditions [2], and although health professionals are trained on attending to these aspects during their studies, their own QoL might decline during their years in medical schools [2].

Many studies have reported decreased QoL scores among medical students during their training years, which is associated with several future adverse effects, including an unhealthy lifestyle, variable psychological problems, academic failure, and other negative impacts on the students’ professional development [3–8]. Various stressors might influence the QoL of medical students, such as a stressful transition from basic to clinical years, continuous demands when interacting with patients, peer competition for academic excellence, the overwhelming load of new and massive information to learn, and of course the difficulty of balancing academic duties with day to day life activities [9–13]. One study in an institute in North America reported that 23% of medical students suffered from depression, while 57% experienced high levels of emotional distress [14]. A similar study from Saudi Arabia found that medical students experienced high levels of psychological distress, with alarming levels of depression, anxiety, and stress [15]. It is also worth noting that medical students were found to suffer from higher levels of stress when compared to students in other programs [16–19], which can affect their QoL. On the other hand, medical students with good physical and mental health are more capable of overcoming the problems within an academic environment [19]. The latter was emphasized in a study in Saudi Arabia, where students who performed better academically, and who demonstrated better QoL scores were, in fact, those of good health [20].

Assessing the QoL of medical students allows for a better understanding of their general condition, and thus, can guide administrations towards specific, context-sensitive, and appropriate interventions to promote students’ QoL. The latter could prevent psychological problems and other pitfalls threatening students’ professional development, and ultimately improve the quality of care provided to future patients [2, 21].

## Methods

This cross-sectional study was conducted among male and female medical students studying at the faculty of medicine at King Abdulaziz University (KAU), in Jeddah, Saudi Arabia, in February 2016. The curriculum of the faculty of medicine at KAU follows a 6-year program, the first year, also referred to as (Pre-med) is an orientation phase, while the second and third years represent the pre-clinical phase and focus mainly on basic sciences. Then, students ascend to the fourth, fifth, and sixth clinical years, where they attend lectures, clinical rounds, and tutorials, as well as some surgical procedures. For this study, we targeted the second-, fourth-, and sixth- year students, and excluded those in the pre-med orientation phase, as well as third and fifth-year students. Second and fourth years represent the start of a new phase in this curriculum, as the second year is the start of the pre-clinical phase, while the fourth year is the start of the clinical phase, and for both phases the following year is considered a continuum of that phase. The choice for the sixth year was made to enhance the scope of this study, since it represents the existing point of these students, and can reflect on their future wellbeing as professionals. All students in the named 3 years were eligible to participate in the study, irrespective of age, gender, or other characteristics. On average, each batch will include about 400 students, making the total of all 3 years 1200 students. Thus, we aimed to include 600 students in this survey, using a convenience sampling technique via approaching students after their morning classes over 2 weeks, and randomly inviting them to participate in this survey. Ethical approval was obtained from King Abdulaziz University’s ethical and technical committee.

The WHOQOL-BREF instrument is a self-administered questionnaire, comprised of 26 items, to assess the four major QoL domains defined by the WHO; physical health, psychological health, social relations, and environment [22]. The first two items separately assess the overall perception of QoL and health. The tool follows a scoring system, where each question is rated on a 5-point Likert scale, ranging from 1 (very poor/very dissatisfied/none/never) to 5 (very good/very satisfied/extremely/always), and then the scores of all four domains are summed and scaled in a positive direction, with higher scores indicating better QoL [22]. Multiple studies have assessed the validity and reliability of the WHOQOL-BREF instrument and acknowledged it as a suitable tool to measure QoL [23, 24]. The Arabic version of the WHOQOL-BREF instrument was also tested and found to be both valid and reliable among Arabic-speaking individuals [25]. Additional questions were added to the questionnaire on demographic data, academic year (i.e., educational level), and Grade Point Average (GPA). The latter was self-reported by students and classified into the following five categories: 2.5 and below to 2.99, 3.0 to 3.49, 3.5 to 3.99, 4.0 to 4.49, and 4.5 and above. As our study was the first to use this Arabic questionnaire for Saudi medical students, we decided to assess the validity and reliability of the WHOQOL-BREF instrument in our sample. To prevent exam stress from influencing responses, data was collected 1 month before the exams. Students were allowed to respond privately in their own time. Participation was voluntary and written informed consent was obtained from all study participants.

The statistical analysis of this study was divided into three parts. First, we tested the internal consistency and reliability of the Arabic questionnaire using Cronbach’s α coefficient and performed a Confirmatory Factor Analysis (CFA) to test its validity. Then, a descriptive analysis of demographic and academic data was carried out, where the scores of each domain were transformed into a linear scale that ranged from 0 to 100, and then represented as means and standard deviations of the total scores. Lastly, the QoL scores for each domain were compared via t-test and one-way analysis of variance (ANOVA), and a significant difference was set at a *P*-value of ≤0.05. We used the IBM Statistical Package for Social Sciences (SPSS) version 21.0 (IBM Corporation, Armonk, NY), and the IBM analysis of a moment structures (AMOS) version 24.

## Results

### Tool reliability

The level of internal consistency for the items of the Arabic WHOQOL-BREF instrument was measured using the Cronbach’s α coefficient, which was 0.867 for the questionnaire as a whole, 0.796 for the physical health domain, 0.755 for the psychological health domain, 0.786 for the social relationships domain, and 0.793 for the environment domain. As a Cronbach’s α coefficient value of > 0.7 was considered a desirable reliability estimate, these results indicated a good internal uniformity for the tested domains.

### Tool’s validity

The construct validity was evaluated through factor analysis using the Kaiser-Meyer-Olkin (KMO) test. The result of the KMO test was 0.911, indicating that data was appropriate. Moreover, Bartlett’s Test of Sphericity yielded a chi-square result of 5584.66, with a significant *p*-value (< 0.001). The latter proved that the sample included in this study was suitable for factor analysis. CFA indicated that all four domains have characteristics roots > 1, and the accumulative contribution rate of 51.2%. Furthermore, the domains’ individual contributions to the variance in QoL were calculated as 32.5, 7.4, 6.1, and 5.2% respectively. Performing CFA revealed an adequate fit for a four-factor model when two matching variables were correlated. X2 = 1378.62, CMIN/DF = 5.60 with *p*-value < 0.001, RMSEA = 0.086, CFI = 0.790, RMR = 0.088, GFI = 0.842, AGFI = 0.808. All item loadings in the questionnaire had a value > 0.3, suggesting adequate construct validity.

### Participants’ characteristics

A total of 630 students responded, where females and males constituted 51.1 and 48.9% respectively. Their mean age was 21.07 ± 1.70 years. Among all respondents, 206 (32.7%) were in the second year, 208 (33%) in the fourth year, and 216 (34.3%) in the sixth year.

**Fig. 1:**
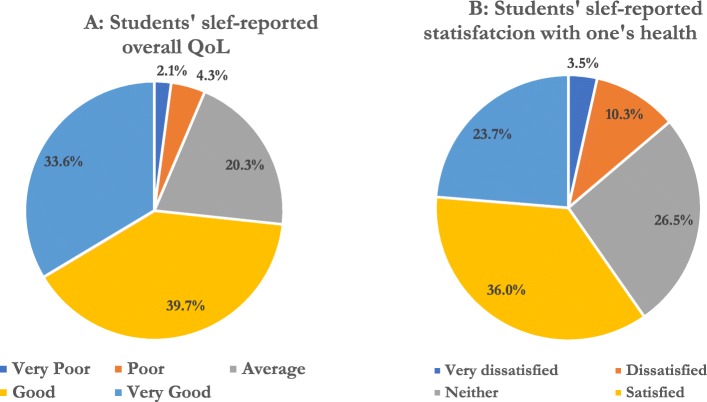
**a** Students’ slef-reported overall Qol. **b** Students’ slef-reported statisfavtion one’s health

Regarding the self-assessment done by the medical students, the mean score of their overall self-reported QoL was 3.99 ± 0.95. In general, 33.6% of the students described their QoL as “very good”, 39.7% as “good”, and only 2.1% felt it was “very poor” (Fig. 1a). On the other hand, the mean score of their self-rated satisfaction with current health was 3.66 ± 1.06. The majority felt satisfied with their health, as 23.7% were “very satisfied” and 36% were “satisfied”, while only 3.5% acknowledged that they were “very dissatisfied” with their health (Fig. 1b).

**Fig. 2:**
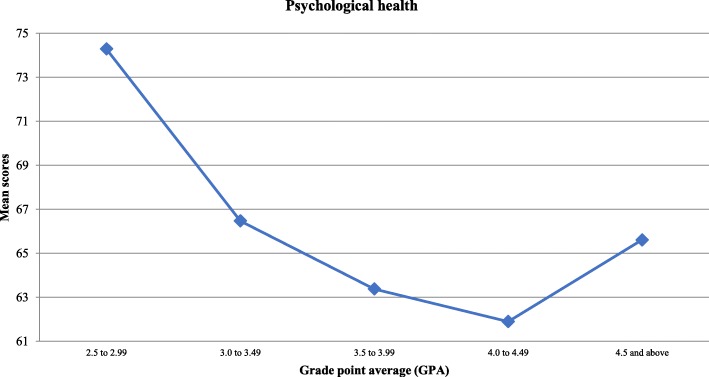
Medical student’s “Psychological Health” scores according to their GPA

**Fig. 3:**
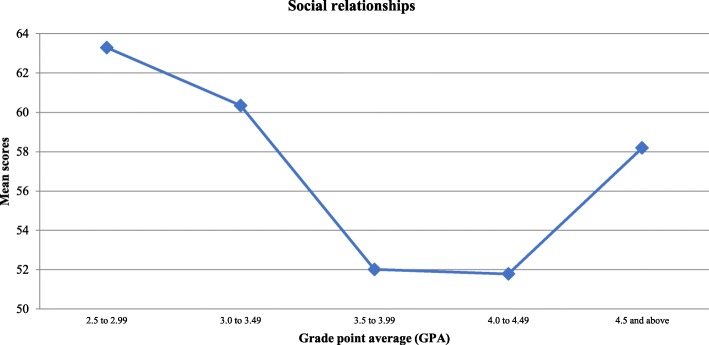
Medical student’s “Social Relationships” scores according to their GPA

With regard to QoL domains and as illustrated in Table 1, the environmental domain had the highest mean score at 67.81 ± 17.39, followed by the psychological health domain at 64.37 ± 14.27, the social relationship domain at 55.67 ± 23.95, and finally the physical domain at 46.94 ± 14.24. When comparing medical students in terms of gender and academic year, the score showed no significant differences across all domains. Yet, students with the lowest GPAs were found to have higher psychological health and social relationships scores. Both findings were statistically significant (*P* < 0.05). The mean scores of both domains in relation to students’ GPA are shown in Figs. 2 and 3 respectively.

**Table 1:** QoL Scores for students per student’s characteristics

Variables	N (%)	Physical Health		Psychological Health		Social Relationships		Environment	
		Mean ± SD	*P*-value	Mean ± SD	*P*-value	Mean ± SD	*P*-value	Mean ± SD	*P*-value
Gender									
Male	308 (48.9%)	46.95 ± 14.57	0.083	64.87 ± 13.81	0.385	55.26 ± 23.95	0.678	67.48 ± 18.67	0.636
Female	322 (51.1%)	44.98 ± 13.87		63.89 ± 14.70		56.06 ± 23.97		68.13 ± 16.09	
Academic Year									
2nd Year	206 (32.7%)	44.87 ± 13.88	0.417	64.08 ± 14.57	0.885	55.95 ± 25.11	0.183	68.34 ± 17.99	0.690
4th Year	208 (33.0%)	46.34 ± 14.60		64.26 ± 14.19		53.36 ± 23.36		66.97 ± 17.39	
Sixth Year	216 (34.3%)	46.58 ± 14.23		64.75 ± 14.11		57.63 ± 23.29		68.12 ± 16.85	
GPA									
2.5 to 2.99	7 (1.1%)	46.57 ± 17.79	0.073	74.29 ± 8.34	0.013	63.29 ± 24.56	0.014	71.57 ± 19.05	0.262
3.0 to 3.49	34 (5.4%)	47.38 ± 13.30		66.47 ± 15.22		60.35 ± 21.04		68.74 ± 18.51	
3.5 to 3.99	82 (13.0%)	47.85 ± 14.79		63.38 ± 14.14		52.01 ± 24.07		66.85 ± 16.76	
4.0 to 4.49	186 (29.5%)	43.45 ± 14.00		61.90 ± 14.14		51.78 ± 23.93		65.68 ± 17.60	
4.5 & above	321 (51.0%)	46.73 ± 14.15		65.61 ± 14.17		58.19 ± 23.88		69.11 ± 17.23

## Discussion

Our results indicate that the Arabic version of the WHOQOL-BREF instrument is both reliable and valid for assessing QoL among medical students in Saudi Arabia. Another major finding of this study was that poor psychological health and social relationships were associated with higher GPAs (in other words, a better academic performance).

Numerous studies worldwide have succeeded in their efforts to validate the WHOQOL-BREF instrument, including a local study that was done among preclinical students in Riyadh [19–21, 23, 24]. In our study, the highest mean score was the environmental domain’s, followed by psychological health, then social relationships, and finally the physical health domain. On a regional level, a study in Pakistan revealed similar results, where the highest reported mean score was that of the environmental domain (70.43), but unlike our study, in this Pakistani study, the psychological health domain had the lowest score 66.5 [26]. Many factors can explain this difference, such as the stable extrinsic environment in Saudi Arabia, both politically and economically, and well-balanced cohesive society supporting students’ psychological wellbeing, relative to the intermittently eruptive context in Pakistan.

Upon exploring factors influencing the students’ QoL, we found that even though students in different years are exposed to different learning environments and workloads, there was no significant difference in the students’ QoL per their academic year. This finding could be attributed to the nature of our curriculum, and the several preparations students underwent before advancing to different levels. These measures challenge them and enhance their readiness for future clinical workload, and they include; problem-based learning (PBL) programs introduced early during the second year, different integrated courses focusing on both basic and clinical aspects of topics, along with an early orientation to clinical training during the first 3 years of medical school. Our findings were consistent with a study among Brazilian medical students in 2015 using the WHOQOL- BREF tool [2]. Yet, after reviewing the literature, we noted that these findings greatly depend on schools’ curriculum and a lower score of QoL was noted across different years depending on the program. For example, in the above study in Pakistan, first year and final year students had the lowest scores in all domains, since these 2 years are the most academically demanding years [26]. On the other hand, another Chinese study revealed that the third-year medical students had the lowest overall QoL scores, and the authors attributed this to the stressful transitions from basic years to clinical years [21].

Interestingly, we found no correlation between the gender of students and their QOL across all domains. This is in contrast with the findings of the above studies. In some studies, males had higher scores in the physical health domain when compared to females [19, 20, 26, 27], whereas in other studies, males showed better psychological health than females [20, 21]. Moreover, one Brazilian study reported that female students had lower scores in most of the domains [27]. Our findings could indicate that despite societal, cultural norms, which impose variations on the modes of living of males and females’ in Saudi Arabia, there is no actual qualitative difference in terms of their QoL.

The most striking result to emerge from the data was that students with the lowest grade point averages (GPAs, 2.5 to 2.99) reported significantly higher scores for both the psychological health and the social relationships domains, and accordingly, those with higher GPAs suffered from poorer psychological and social wellbeing. Among the plausible explanations for this is that better-achieving students are under much pressure to continuously improve and sustain their academic advancement, unlike those who perform poorly, and are neither interested in peer competition nor in acquiring high marks. The latter might have allowed them to spend more time socializing, and engaging in leisure activities, and thus boosted their psychological and social wellbeing health, as was noted elsewhere [28]. Yet, this contradicts with the results of another study conducted in Saudi Arabia, where medical students with better academic performance reported higher scores in all QOL domains [20]. Similarly, a study in the United States concluded that students with higher GPAs are physically healthier, in comparison to those with less academic achievements [29]. There is a need to install an efficient and appropriate student support system, especially for better-performing students who suffer from huge stress during their studies.

We believe our study to be the first to evaluate the effect of gender, educational level, and academic performance, on the QoL among both pre-clinical and clinical medical students in Saudi Arabia. However, there were some unavoidable limitations. First, the sample was selected from only one medical school, meaning that the results might not represent all medical students in Saudi Arabia. Therefore, further research should be undertaken involving a larger sample across different medical schools. Second, self-reporting methods were used, and no verifications were requested for the students’ GPAs. Finally, despite using the WHOQOL-BREF instrument to assess students’ QoL, using comprehensive qualitative techniques could yield more accurate results, and offer a better understanding of the students’ perspectives.

## Conclusion

The WHOQOL-BREF is a valid and reliable instrument for assessing QoL among Saudi medical students. Gender and educational level did not affect medical students’ QoL, but high-achieving students reported lower QoL scores, possibly because of the stress they are under. Efficient support systems are needed to accommodate these students.

## Data Availability

The datasets generated in the study are not publicly available due to individual privacy issues but are available from the corresponding author on reasonable request.

## Abbreviations

GPA: Grade Point Average
QoL: Quality of Life
